# 构建中国血液学共同体

**DOI:** 10.3760/cma.j.cn121090-20240523-00186

**Published:** 2024-06

**Authors:** 晓军 黄, 郑丽 徐

**Affiliations:** 北京大学人民医院，北京大学血液病研究所，国家血液系统疾病临床医学研究中心，造血干细胞移植治疗血液病北京市重点实验室，北京 100044 Peking University People's Hospital, Peking University Institute of Hematology, National Clinical Research Center for Hematological Diseases, Beijing Key Laboratory of Hematopoietic Stem Cell Transplantation for the Treatment of Hematology, Beijing 100044, China

**Keywords:** 中国, 血液学, 共同体, China, Hematology, Community

## Abstract

中国血液学的发展自20世纪50年代逐渐拉开序幕，历经几代人的艰苦奋斗、薪火相传，已成长为一个年轻但充满活力的学科。中国血液学的发展经历了快速崛起和稳步成长，但与国际血液学发展水平仍有差距。只有通过构建中国血液学共同体，形成推动血液学发展的合力，才能更好地实现血液学领域的中国梦。

## Abstract (en)

The development of hematology in China has gradually begun since the 1950s. After several generations of hard work, it has grown into a young but vibrant discipline. The development of Chinese hematology has experienced rapid rise and steady growth, but there is still a gap with the international level of hematology development. Only by constructing a Chinese community of hematology and forming a joint force to promote the development of hematology can we better realize the Chinese Dream in the field of hematology.

中国血液学的发展自20世纪50年代逐渐拉开序幕，历经几代人的艰苦奋斗、薪火相传，已成长为一个年轻但充满活力的学科。从国际上最早发现急性髓系白血病（AML）M_2b_亚型到亚洲第1例异体骨髓移植，从上海方案到北京方案，都彰显着中国血液学的快速崛起和稳步成长。但同时我们也应正视与国际血液学发展的差距，只有通过构建中国血液学共同体，形成推动血液学发展的合力，才能更好地实现血液学领域的中国梦。

一、峥嵘岁月，历史突破奠定发展基础

我国血液学前辈们深耕科学研究，拓荒学科空白，在白血病诊疗、造血干细胞移植和血栓性疾病等领域开创了历史先河。1959年，中国医学科学院血液病医院杨崇礼教授等首次定义了一种新的白血病类型——亚急性粒细胞白血病，随后按照FAB分型原则定义为AML-M_2b_，该类型白血病的发现比国外早了12年。1964年北京大学人民医院陆道培院士完成亚洲第1例、全球第4例造血干细胞移植术，开启了中国骨髓移植史的序章，随后在1981年完成中国首例异基因骨髓移植。1983年苏州大学附属第一医院阮长耿院士成功培养出我国第一组抗人血小板膜糖蛋白单克隆抗体，自1987年起，其中5株抗血小板单抗通过国际分化抗原委员会鉴定，成为血小板研究的标准试剂。1986年，上海交通大学瑞金医院王振义院士首次应用全反式维甲酸治疗急性早幼粒细胞白血病（APL）患者并实现疾病治愈，成为全球公认的诱导分化理论的第一个成功案例。

二、守正创新，中国原创方案引领世界

21世纪以来，我国血液学研究者继往开来、勇于探索，创建了国际公认的中国原创方案，其中上海方案是靶向治疗的先驱，北京方案是中国原创技术改变世界格局的典范。

上海交通大学瑞金医院王振义院士、陈竺院士、陈赛娟院士等创造性地提出“全反式维甲酸联合三氧化二砷”治疗APL，创建了协同靶向治疗的理论体系，并实现了临床转化的重大突破，使APL成为第一个可基本治愈的AML[1]。该突破性成果被誉为治疗APL的“上海方案”，现已被全球广泛应用。

2000年北京大学人民医院黄晓军院士首创粒细胞集落刺激因子（G-CSF）动员的供者干细胞采集物联合受者应用抗胸腺细胞球蛋白（ATG）的临床免疫耐受新方案，应用该方案成功完成首例非体外去T细胞单倍型相合造血干细胞移植手术。随后，黄晓军院士带领团队创建系列技术，形成全球首个单倍型相合移植体系，2016年世界骨髓移植协会主席把这一系统性工作称为“北京方案”[2]。“北京方案”的关键技术推广到全国190余家造血干细胞移植中心及意大利、以色列、法国、韩国等多家海外移植中心，推动了全球造血干细胞移植的快速发展[3]–[4]。

三、百花齐放，紧跟国际潮流与之共舞

近年来，精准诊断及免疫、靶向、基因等新型治疗策略的突起正悄然改变着血液病学的诊疗模式，我国血液学研究者在精准诊疗领域紧跟国际潮流，如细胞治疗、基因治疗和靶向药物等，在世界血液学舞台奏响华夏乐章。

我国嵌合抗原受体T细胞（CAR-T细胞）治疗血液恶性肿瘤在过去10余年取得了显著进步：2013年进行第1次CAR-T细胞临床试验，2017年中国成为世界上CAR-T细胞临床研究数量最多的国家。中国CAR-T细胞治疗在相关靶点探索和临床疗效提升方面取得了卓越成果，其中发展创新聚焦于新型靶点、功能强化、精准调控、通用型CAR-T细胞研发及结合干细胞定向分化技术等方面[5]。

我国在基因治疗领域的基础和临床研究方面不断取得飞跃，2019年第1例血友病B患者的入组标志着亚洲首个以肝脏靶向腺相关病毒（AAV）为载体血友病B基因治疗研究的开启[6]。该研究对于推动我国基因治疗药物研发和临床转化具有重要的指导意义。

我国首个自主研发的口服第三代BCR-ABL抑制剂奥雷巴替尼于2021年11月获得中国国家药品监督管理局（NMPA）的批准。奥雷巴替尼的开发和上市标志着中国在血液肿瘤靶向治疗领域的重要进展，是血液病领域中国创新药物研发的一个典范[7]。

四、正视差距，齐力构建血液学共同体

如前所述，我国在血液病诊疗方面已取得系列重要成果，在造血干细胞移植、靶向治疗等领域处于国际先进水平，在精准诊断、细胞治疗、基因治疗等新兴领域也得到快速发展，具有一定的国际影响力。然而，我们也应正视我国血液病整体诊疗水平与国际水平的差距，未来需在规范化治疗、新药研发、技术创新等方面持续努力。

1. 原创诊断体系匮乏：从血液肿瘤诊断体系来看，目前白血病、骨髓瘤、淋巴瘤等诊断和分层均以国际标准如美国国立综合癌症网络（National Comprehensive Cancer Network，NCCN）指南为参考基础。以APL为例，我国多个团队研究发现APL新的融合基因，如TBLR1-RARα、CPSF6-RARG、STAT3-RARA、NPM1-RARG-NPM1[8]–[11]，但上述新融合基因的发现仅是对APL诊断体系的扩充，APL诊断和分层预后体系仍源自国际标准[12]。未来以期通过基础研究深入探索与血液肿瘤发生发展相关的新机制和新靶点，为原创诊断体系提供科学依据。

2. 原研药物和技术欠缺：聚焦于血液系统疾病治疗领域，我国在原研药物开发和原创技术开展方面与国际水平仍有差距。AML小分子靶向药物如FLT3、IDH1、IDH2、Bcl-2抑制剂，急性淋巴细胞白血病免疫治疗如单抗、双抗等，均由欧美国家研发并获批上市，在欧美国家的临床应用较为广泛。相比之下，中国在这些药物的原研开发上起步较晚，药物的上市时间也相对落后。原创技术方面以CAR-T细胞治疗为例，虽然中国在CAR-T细胞治疗的靶点创新、技术改造和优化方面取得了一定的进展，但目前中国批准上市的CAR-T细胞产品主要通过技术引进和合作生产的模式，尚未形成完全自主的原创制备技术。

3. 规范化诊疗水平差异大：我国不同地区的医疗资源分布不均，一线城市和发达地区的诊疗水平通常较高，而一些基层医院或偏远地区的诊疗水平相对较低，这种差异导致患者接受的诊疗服务和治疗效果存在较大差别。血液系统恶性疾病的种类繁多，每种疾病又有多种亚型，精确的分型对于制定个性化治疗方案至关重要，但基层医疗机构专业人才相对不足、精确分型尚难推广。此外，虽然我国已经制定了一系列血液肿瘤的诊疗规范，但在实际执行过程中，由于各种原因如医疗资源限制、经济因素等，诊疗规范的执行力度参差不齐。

为了缩小与国际血液学领域的差距，我们需要进一步凝心聚力，加强学术交流与合作，促进资源共享和优势互补；通过协同创新加快血液学领域的科研成果转化，提升诊疗技术水平，更好地服务于广大患者。构建中国血液学共同体是实现这一目标的重要途径，而构建中国血液学共同体需在以下几方面努力。

（1）促规范：通过建设协作组与大数据联盟，统一制定和推广临床规范、指南及临床路径，形成行业规范或标准，提高治疗的标准化和同质化水平。2021年由国家血液病临床研究中心、北京大学人民医院牵头发起的，以“促规范·鉴未来”为主题的中国血液疾病规范诊疗中心协作体启动。“协作体”通过建立规范化诊疗教学示范基地、多学科交流中心、各中心联动定期协作会议等多种方式打造多维度立体化的学术交流创新平台，以点带面、共同促进血液系统疾病规范诊疗协作。

（2）求创新：创建临床诊疗新方案，实现血液病诊治的原始突破是提高我国血液系统疾病诊疗水平的关键。鼓励采用新技术、新方法，如新型诊断技术、基因编辑技术、细胞治疗技术等，探索血液病诊疗的新途径。加大对血液病新药研发的支持力度，鼓励创新药物的研发，以满足实际临床需求。要实现上述目标就要创新，不是依赖一个或若干个单位的创新，而应该发挥体制优势协同创新。发挥体制优势，促进医疗机构、科研院所、高等学校和企业之间的合作，形成创新联合体。

（3）立转化：在血液系统疾病基础研究和临床转化方面，我国还需要进一步加强。如加大基础研究投入，加强血液系统疾病的基础研究，包括疾病的分子机制、新型药物靶点的发现等。在医疗机构和科研院所中建立转化医学研究中心，专注于将基础研究成果转化为临床应用。建立多学科协作平台，促进生物学家、药理学家、临床医师等不同领域专家之间的交流与合作。以此提高基础研究与临床应用的转化效率，最终促进科研成果的临床转化应用。

（4）重合作：加强与国际先进医疗机构的交流与合作，引进和学习国际先进的诊疗技术和经验。在全球化背景下，国际合作与竞争对新药研发具有重要影响。我国需要进一步加强与国际先进水平医疗机构的合作与交流，建立国际合作平台，促进我国与国际医疗机构在血液病领域的合作；引进先进技术，培养高水平的研发人才，提升自主创新能力；参与或发起国际多中心临床试验，提高临床研究的质量和效率等。

“路远行则至，事难做必成”。尽管目前我国血液学诊疗水平与国际水平存在一定差距，但我们希望通过构建中国血液学共同体形成推动发展的合力，逐渐缩小与国际水平的差距。中国血液学研究者应以更开放的胸怀、更高远的格局团结协作，为推进我国血液病的规范化诊疗建设做出贡献。中国血液学共同体将通过建立一个开放、合作、共享的平台提升中国血液学的国际影响力，让中国的血液学研究和治疗水平达到国际先进水平，为人类健康事业作出更大的贡献，为实现中华民族伟大复兴贡献血液学工作者的力量。
